# Prevalence of adolescent obesity at a high school in the City of Tshwane

**DOI:** 10.4102/curationis.v40i1.1662

**Published:** 2017-05-23

**Authors:** Nomusa A. Ngwenya, Tendani S. Ramukumba

**Affiliations:** 1 Adelaide Tambo School of Nursing Science, Tshwane University of Technology, South Africa

## Abstract

**Background:**

Obesity has been reported to be on the rise in the world and South Africa is no exception. In recent years obesity has been reported to contribute to the increasing number of people with hypertension and diabetes mellitus. Africa has the fastest growing number of overweight and obese adolescents, with indications that in 2010, it had more than doubled since 1990. Some people might perceive being overweight as being round and healthy which might contribute to the increased rate of obesity in South Africa. Physical and psychological changes that occur during adolescence can also be observed earlier during the preteen years (ages 9–12 years). During this time, peer groups and external appearance are of importance. Physical changes, such as obesity, might be perceived negatively by adolescents, affecting their self-esteem.

**Objectives:**

The purpose of the study was to determine the prevalence of adolescent obesity at a high school in the City of Tshwane.

**Method:**

A cross-sectional survey was conducted. Stratified random sampling was used and data were collected from 30% of the total population as recommended by the statistician. Data analysis was performed using descriptive analysis. Validity and reliability were ensured through calibrating the weight-monitoring scale and the measuring tape, which are collection tools.

**Results:**

The results identified the prevalence of adolescent obesity at 8.57%. There is evidence of abdominal obesity and waist–hip ratio ≥ 1. The results show that there is a steady increase in obesity levels among adolescents. The poor response of parents was observed and could demonstrate the need to form stronger partnerships for weight reduction interventions.

**Conclusion:**

Evidence-based prevalence allowed for conceptualisation of the scope of the obesity epidemic and how children and young people are also affected. To enable proper planning for adolescent obesity interventions, the depth of consequences of obesity for the adolescent cohort should be well defined and clarified.

## Introduction and literature review

According to Ogden et al. (2014:806), overweight is defined by a body mass index (BMI) above 25 kg/m^2^, while obesity is characterised by a BMI above 30 kg/m^2^. In addition obesity is a term used mostly to describe people with excess body weight. According to Krebs et al. (2011:S193), obesity is defined as excess fat in the body. De Onis, Blossner and Borghi (2010:1257) reported that Africa has the fastest growing number of overweight or obese adolescents, with indications that in 2010, the incidence of obesity had more than doubled since 1990. Armstrong, Lambert and Lambert (2011:439) compared the prevalence rates from the South African primary schools’ anthropometric survey and the health of the nation study. These authors estimated an increase in overweight from 1.2% to 13% and in obesity from 0.2% to 3.3% over the period 1994–2004. The estimated increase of obesity might be a risk factor for most adolescents and the leading causes of morbidity and mortality including cardiovascular diseases, diabetes, strokes and some cancers. Specifically abdominal obesity is associated with diabetes and coronary diseases (Stafford et al. 2007:1884). These obesity-related diseases can be curbed among adolescents through obesity prevention.

According to Pickhardt (2012), adolescence is the years between the ages of 13 and 19, which is regarded as the transitional stage from childhood to adulthood. Physical and psychological changes that occur in adolescence can also be observed earlier during the preteen years (ages 9–12 years). During this time, peer groups and external appearance are of importance, and if negatively perceived physical changes occur, such as obesity, the teens’ and preteens’ self-esteem might be affected negatively. According to Schwarz and Peterson (2010), adolescence is a crucial period for establishing healthy behaviours. However, according to Farhat, Iannotti and Simons-Morton (2010:258), obese adolescents are particularly vulnerable to health-related risk behaviours and are more likely to demonstrate maladaptive coping, such as overeating or eating the wrong foods. Compared to their normal weight peers, obese adolescents are more likely to experience impaired peer relationships, stigmatisation and weight bias. Instances when maladaptive behaviours are not well managed might lead to wrong eating behaviours and obesity among adolescents.

According to Stafford et al. (2007:1884), there is a complex picture of significant facilitators of obesity among adults, adolescents and children. These facilitators are referred to as the contributing factors towards adolescent obesity, encompassing influences and interactions that include genetic, behavioural and environmental factors. Knowledge, perceptions and attitudes are facilitators of obesity in adolescents as they influence the way decisions are made.

Stafford et al. (2007:1884) further associated obesity in children with the safety of the environment in which the children and their parents feared exposure to danger. This fear facilitates a lack of physical activity among children and promotes indoor activities which are carried out with limited physical activities, resulting in the imbalance between energy intake and energy expenditure, aggravating these persons’ potential to become obese. Other factors leading to increased obesity among adolescents include interpretation of obesity as a sign of financial and well-being status. Wallace (2006:129) stated that African-American adolescents associate excessive weight with being a breadwinner and with the ability to afford luxury foods such as meats and fried foods. In addition a study conducted by Puoane et al. (2005:10) revealed that participants in Cape Town perceived being overweight as being round and healthy which influenced the increased rate of obesity. This perception might have encouraged obesity among adolescents while establishing their identity.

### Problem statement

Obesity in the global community, including South Africa, has been observed to be on the increase based on anthropometric measurements among school children. The researchers, who provide school health services, have observed an increase in obesity among the adolescents attending a selected school in Tshwane. The main research problem was that the prevalence of obesity among adolescents in this high school was unknown. Such knowledge could help to formulate guidelines for effective interventions to address obesity-related issues in this school.

## Aims of the study

The purpose of the study was to determine baseline data for designing and implementing future obesity-related intervention programmes. The other purpose was to establish the prevalence of adolescent obesity. The aim of the research captures the essence of the study including the variables and population (Brink, Van der Walt & Van Rensburg 2012:59).

## Research setting

The study was contextual as it was conducted at one specific high school in the City of Tshwane. A contextual study is a study that is conducted at a specific setting, and the results can only be applied to that setting (Burns & Grove 2009:696). The school was opened in 1955. The school is situated on the banks of the Apies River. The City of Tshwane had a population of 2 921 488 according to the 2011 census (Statistics South Africa 2011). The main languages spoken in the City of Tshwane are Sepedi, Afrikaans, Setswana, Xitsonga, isiZulu and English. IsiNdebele and Sesotho are also widely spoken. The high school is a commuting school with 954 children commuting from different parts of the Tshwane Metropolitan area.

## Research objectives

The research objective was to determine and describe the prevalence of obesity among adolescents at a high school in the City of Tshwane.

## Definition of key concepts

*Prevalence*: Refers to the proportion of individuals in a population having a disease or characteristic (Zwiegenthal et al. 2009:95). A number of adolescents at this particular high school who are obese make the prevalence value.

*Adolescent:* The WHO (2015:2) defines adolescence as the transition period in human growth and development that occurs after childhood and before adulthood, from ages 10 to 19. In this study the adolescent was a person aged 13–19 years old.

*Adolescent obesity:* Adolescent obesity in this study was characterised by a BMI above 30 kg/m^2^; in addition it is a term used mostly to describe adolescents with excess body weight (Reilly et al. 2003:749).

## Contribution to field

The results of the study indicated that the prevalence of adolescent obesity was at 8.57%. Prevalence allows planning for adolescent obesity interventions such as an age-related physical activity and nutritional programme. The programme would enable decision-making and raise awareness of adolescent obesity.

## Research method and design

### Design

According to Burns and Grove (2009:695), cross-sectional designs are used to examine groups of subjects in various stages of development simultaneously with the intent of inferring trends over time. The cross-sectional survey adopted in the current study assisted in determining the prevalence of adolescent obesity at a high school in the Tshwane municipal area.

### Population and sampling

According to Brink et al. (2012:123), the population is a group of people that are of interest to the researcher.

- *Target population*. The target population comprised 954 scholars at the participating high school. According to Burns and Grove (2009:343), a target population is defined as the entire set of individuals who meet the sampling criteria.
- *Accessible population*. According to Brink et al. (2012:123) accessible population is the population that a researcher has access to as it is usually rare for the researcher to access the entire population. The accessible population in the study was adolescent scholars at the high school who were available and willing to participate.

#### Sampling method and size

Stratified random sampling was used. According to Brink et al. (2012:130), in stratified random sampling the population is divided into subgroups according to the variables of importance in the study. Stratified random sampling further provided the advantage of representation of particular segments of the population. The statistician consulted recommended 30% of the target population as a sample size, which implied 286 participants should be included. However, in the end only 175 scholars were willing to participate. Though the targeted sample size was 286, a total of 450 invitation leaflets were distributed to the scholars inviting 225 males and 225 females to participate in the study. Only 175 scholars responded and participated, resulting in 61.2% of the targeted population and consequently 39% response rate of the 450 invitees.

### Data collection method

Data collection method was through observation. An anthropometric assessment was conducted including measuring of body weight, height, waist circumference and hip–waist ratio (Burns & Grove 2009:396) as shown in Table 1. A checklist was developed to gather the demographic information of the participants and record the measurements. The data collection tool’s validity was ensured as it was used in another study (Ramukumba 2012:412). Data collection equipment were a calibrated weight scale and a measuring tape.

**TABLE 1:** Grade participation.

Grade	Frequency	Percentage	Cumulative %
8	23	13.14	13.14
9	17	9.71	22.86
10	37	21.14	44.00
11	52	29.71	73.71
12	46	26.29	100.00
**Total**	**175**	**100.00**	**-**

Participants’ age (*N* = 175).

Data were collected during an allocated Life Orientation period which was arranged by the principal of the school. As per the condition of Gauteng Basic Education Department, minimal disruptions of the school programme had to be achieved.

### Data analysis

Statistical analysis was performed using Stata v12 (Stata Corporation) statistical software package. Categorical variables were summarised through frequency tables and the mean, standard deviation, minimum and maximum scores were used to summarise the continuous variables. Chi-square tests were performed to determine whether there was a significant relationship between gender and weight status. According to Brink (2006:171), descriptive statistics imply a method inclusive of frequency distributions, measures of central tendency and variability.

### Ethical consideration

The Departmental Research and Innovation Committee and Research Ethics Committee of the Tshwane University of Technology approved the protocol.

The Gauteng Department of Basic Education Research Committee approved the protocol and granted permission to conduct the study.

## Results

This section provides a synthesis of the results obtained, grouped according to the subsections of the topic of interest, namely the prevalence of obesity, abdominal obesity and waist–hip ratio among school children in Tshwane.

### Demographic data analysis

Out of the 175 participants (*N* = 175), 62.86% (*n* = 110) were female and 37.14% (*n* = 65) male. Participants were spread according to grades, from Grades 8 to 12. Table 2 reflects the scholars’ participation according to the grades at the school.

**TABLE 2:** Variables.

Variable	Population	Results	
		Normal	Abnormal
Waist	175	166	9 = 5.14%
Waist–hip ratio	175	171	4 = 2.28%

The younger groups of participants which are 13-and 14-year- olds had the lowest response as their assent was not completed and returned, while among the older group the level of participation was higher.

### Body mass index

Figure 2 presents a graphical presentation of the participants’ BMI indices and the significant relationship between gender and BMI status using a *t*-test. After the *t*-test was performed to test the significant relationship between gender and BMI status, the *p*-value was 0.290. There was no significant relationship between gender and BMI.

**FIGURE 1:**
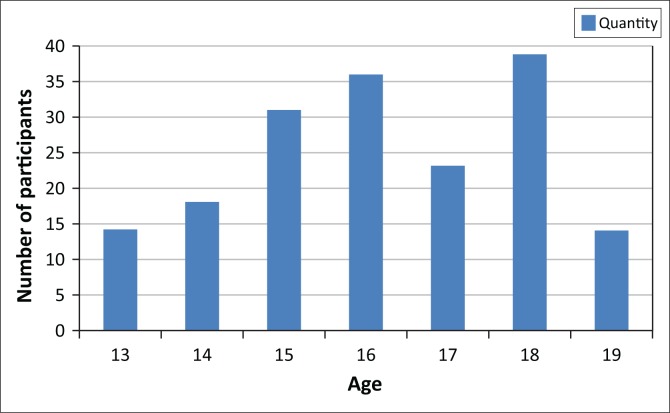
Participants’ age.

**FIGURE 2:**
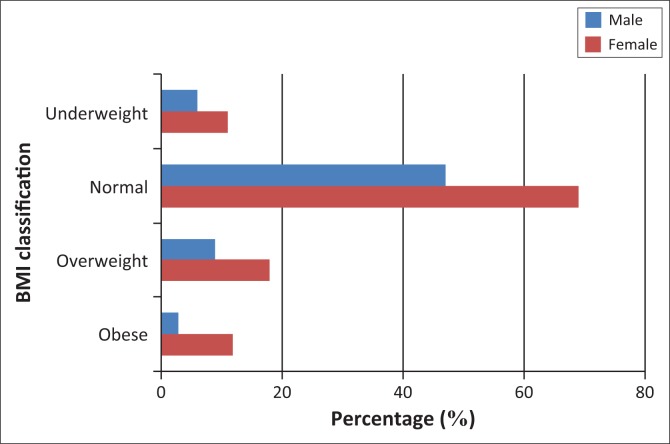
BMI analysis and gender relationship.

The study found that the average BMI was 23.5 kg/m^2^, the minimum was 14.8 kg/m^2^ and the maximum BMI was 43.57 kg/m^2^. The analysis also showed that of the sample population, 9.71% (*n* = 17) were below 18 kg/m^2^ which is underweight, 66.28% (*n* = 116) were between 18 kg/m^2^ and 25 kg/m^2^, while 15.43% (*n* = 27) were between 25.1 kg/m^2^ and 30 kg/m^2^ placing them in the overweight bracket. Participants who were found to be obese were 8.57% (*n* = 15) with a BMI above 30 kg/m^2^.

### Abdominal obesity

Abdominal obesity was identified in 5.14% of the participants (*n* = 9). Eight (*n* = 8) female participants were with a waistline of above 88 cm, and one (*n* = 1) male participant was with a waistline of above 102 cm. All identified participants with abdominal obesity had a BMI of above 25 kg/m^2^. Only four participants (2.28%) were with a waist–hip ratio ≥ 1.

### Potential benefits and hazards

There was no harm intended by the study or the findings of the study to the participants and their environment.

The measurements taken were not invasive or painful. Participants were informed that should they get upset during the physical measurement, the researcher and research assistant would have been able to refer such persons to appropriate health care services. However, this was never necessary.

### Recruitment procedures

The choice of the research site fairly took place because the school chosen was a commuting school representing not only a closed community but various communities in the City of Tshwane.

Participation in the study was voluntary, and scholars were allowed to terminate their participation at any time.

Autonomy: Participants were given information leaflets, which stipulated their right to participate and emphasised that they could withdraw from the study at any stage. The letter of informed consent explained the procedures, the duration of the study and the role of participants during the study and the benefits of participating in the study.

### Informed consent

Permission to conduct the study was requested from and granted by the Gauteng Department of Basic Education (2014/276), the School Governing Body and the principal of the school. Consent and assent were obtained from the participants and their parents.

### Data protection

Anonymity was ensured because participants’ names were not used, instead a coding system was used when data were analysed and reported. The name of the school was masked to protect the school from potential negative stigmatisation.

Confidentiality was maintained because information obtained from the participants was kept confidential throughout the study. Only the researchers and the statistician had access to the raw data.

A nursing student was recruited as a fieldworker and was trained by the researchers about the importance of confidentiality to ensure proper handling of the data. A confidentiality agreement, as stipulated by the Innovation Committee and Research Ethics Committee of the Tshwane University of Technology, was signed by the researchers and the fieldworker prior to participating in the study.

### Reliability and validity

Reliability refers to the accuracy and consistency of the information obtained in the study, and validity is the ability of the instrument to measure the research variables as intended (Polit & Beck 2008:196). The reliability in the study was enhanced through ensuring that data collected were accurate by training the fieldworker, and closely supervising the data collection process. Physical measuring instruments were calibrated. The weighing scale was calibrated to ensure accuracy of findings. Stature measurement was performed using a measuring tape using centimetres. Hip and waist were also measured in centimetres using measuring tape.

## Discussion

### Outline of the results

The results of the study will be discussed as follows:

### Grade participation

The response rate was 61.2% of the targeted population. The results reflected that there was no fair grade distribution when it came to the grade participation. The higher grades had a better response as the Grade 11 group had more participants at 29.71%, with Grade 12 at 26.29%. In a study conducted by Vanhelst et al. (2013:2) of 573 eligible parents, 261 returned the completed questionnaires, which also recorded a low return; this was attributed to the fact that the parents perceived that there were no direct benefits for their children. In this current study, the low return which was evident in the lower age category could also be explained using the Lasagna’s law. Lasagna’s law is a phenomenon coined by the clinical pharmacologist Louis Lasagna, who stated that researchers in health overestimate the number of patients available for a research study resulting in recruitment of a fraction of participants as assumed before the study (Van der Wouden et al. 2007:8190). Most of the Grade 9 group reportedly forgot their consent forms at home and some reported that their parents did not sign the consent forms, and only 9.71% brought their forms back signed. Literature on parents’ involvement is silent about involvement related to school health-related projects (Hill & Tyson 2009:743). When comparing the grade participation it is safe to conclude that the participants in the higher grades were easier to recruit because some were able to sign their own consent as they had reached the age of 18 when they can legally sign consent in South Africa.

### Body mass index analysis and findings

Tzioumis and Adair (2014:231) found that the burden of malnutrition still poses a threat to children’s health in countries with lower and middle income. Under-nutrition and over-nutrition coexisted among the scholars who participated in the current study. WHO (2016) also stated that it is common to have under-nutrition, overweight and obesity existing side by side within the same community settings. Furthermore, this double burden is exposure to high-fat, energy-dense, micronutrient-poor foods and a lack of physical activity as the children grow into adolescence. Hence, it has become necessary to continually explore the prevalence of adolescent obesity to enable awareness programmes in different settings. The prevalence of obesity in the current study was 8.57% while overweight was 15.43%. The findings concur with the study conducted by Reddy et al. (2010:43) in which the findings of overweight are 19.7%, and obesity at 5.3%.

Obesity trends are causing public health concerns globally, and they paint a picture of serious consequences when those children become adults. The risk factors associated with obesity are early onset of cardiovascular diseases, increased mortality and morbidity (Raj &Kumar 2010: 598). Many co-morbid conditions such as metabolic syndrome, orthopaedic with backache and poor movement, neurological such as peripheral neuropathy and headache, liver diseases, pulmonary and renal disorders are also seen in association with childhood obesity (Sahoo et al. 2015:189).

Abdominal obesity was also detected in the study conducted by Silva et al. (2011:78), who stated that abdominal obesity was among the important risk factors for acute myocardial infarction in adults, extending to adolescents as central fat has revealed an association with increased risk of cardiovascular diseases during adolescence and early adulthood. Sahoo et al. (2015:189) assert that childhood obesity can affect children’s physical health. In addition, the effect also includes social, emotional well-being and self-esteem. Furthermore, it is also associated with poor academic performance and a lower quality of life experienced by the scholar (Kolotkin 2006:448).

Excess intra-abdominal adipose tissue accumulation, which is also visceral obesity, forms part of phenotypes including dysfunctional subcutaneous adipose tissue expansion and ectopic triglyceride storage (Tchernof & Després 2013:363). This is related to clustering cardiometabolic risk factors leading to the inflammatory process responsible for hypertension and its complications, type II diabetes mellitus and its complications including all carcinogenic reactions associated with obesity. All these lead to a web of complications which is costly to treat. Hence, the researcher suggests decisive weight reduction programmes aimed at curbing the growing trend of obesity among children and adolescents.

The waist-to-hip (WTH) ratio is a common measure of fat distribution (WHO 2011). Your WTH ratio can help you track your weight loss progress, while also serving as a warning about your estimated health risk for problems related to being overweight, such as diabetes, stroke and heart disease.

## Practical implications

The research findings are to be used in raising awareness, highlighting the importance of prevalence results including planning for adolescent obesity interventions in schools to reduce the rise of obesity.

## Limitations of the study

The time allocated for data collection was inadequate as minimal disruptions of the school programme had to be achieved. The time allocated was during an allocated Life Orientation period which was arranged by the principal of the school. Scholars who reportedly forgot their consent forms and those whose parents reportedly did not sign the forms contributed to the relatively low response rate. Scholars’ fear about the study might have limited their responses. The findings are based only on the calculated BMIs of the participating scholars. Such additional information could have enabled the identification of individual scholars who could have been referred to specific health care facilities for interventions.

## Recommendations

### Research

Further studies need to be conducted to establish facilitators of adolescent obesity and risk factors associated with obesity in adolescents. Future researchers should implement interventions to reduce the prevalence of obesity and evaluate the outcomes of such programmes. Future studies could yield worthwhile findings if the scholars’ BMIs could be correlated with their diets, levels of physical activity and any potential health-related issues such as diabetes or hypertension or possible orthopaedic problems.

### Nursing practice

The integrated school health programmes should be accessible to all schools. If high rates of obesity exist at any school, appropriate interventions should be offered to such a school’s learners. Obesity-related health education should also be provided by school health nurses on a regular basis.

### Conclusion

Obesity remains a serious health threat in the South African society, and diverse strategies are required to deal effectively with the rising obesity prevalence. Obesity poses health-related risks to scholars. These risks could be reduced, and scholars’ quality of life could be enhanced if successful interventions could be provided to schools where the obesity prevalence is high. However, the coexistence of cases of under-nutrition and obesity among the scholars needs to be addressed using appropriate nutritional interventions.

## Consequences

### Physical

Obesity is associated with a higher risk for the development of insulin resistance, type 2 diabetes mellitus and a number of cardiovascular abnormalities during childhood and adolescence, as well orthopaedic problems due to excessive weight bearing (Rossouw, Grant & Viljoen 2012).

### Emotional

Overweight and obesity during adolescence can give rise to a lack of confidence, negative self-perceptions and depression (Rossouw et al. 2012). As obesity might limit physical activities, the situation might get progressively worse for individual children if no effective interventions take place.

## References

[CIT0001] Armstrong M.E.G., Lambert M.I. & Lambert E.V, 2011, ‘Secular trends in the prevalence of stunting, overweight and obesity among South African children (1994–2004)’, *European Journal of Clinical Nutrition* 65, 835–840. 10.1038/ejcn.2011.46 21505505

[CIT0002] Brink H., Van der Walt C. & Van Rensburg G, 2012, *Fundamentals of research methodology for health professionals*, 3rd edn., Juta, Cape Town.

[CIT0003] Burns N. & Grove S.K, 2009, *The practice of nursing research: Appraisal, synthesis, and generation of evidence*, 6th edn., Saunders, Philadelphia, PA.

[CIT0004] De Onis M., Blössner M. & Borghi E, 2010, ‘Global prevalence and trends of overweight and obesity among preschool children’, *American Journal of Nutrition* 92(5), 1257–1264. 10.3945/ajcn.2010.29786 20861173

[CIT0005] Farhat T., Lannotti R.J. & Simons-Morton B.G, 2010, ‘Overweight, obesity, youth, and health-risk behaviours’, *American Journal of Preventive Medicine* 38(3), 258–267. 10.1016/j.amepre.2009.10.038 20171527 PMC2826832

[CIT0006] Hill N.E. & Tyson D.F, 2009, ‘Parental involvement in middle school: A meta-analytic assessment of the strategies that promote achievement’, *Development Psychology* 45(3), 740–763. 10.1037/a0015362 PMC2782391 19413429

[CIT0007] Kolotkin R.L., Zeller M., Modi A.C., Samsa G.P., Quilan N.P., Yanovski J.A. et al., 2006, ‘Assessing weight-related quality of life in adolescents’, *Obesity (Silver Spring)* 14(3), 448–457. 10.1038/oby.2006.59 16648616 PMC2374918

[CIT0008] Krebs N.F., Himes J.H., Jacobson D., Nicklas T.A., Guilday P. & Styne D, 2011, ‘Assessment of child and adolescent overweight and obesity’, *Paediatrics* 120(4), S193–S228. 10.1542/peds.2007-2329D 18055652

[CIT0009] Ogden C.L., Carroll M.D., Kit B.K. & Flegal K.M, 2014, ‘Prevalence of childhood and adult obesity in the United States, 2011–2012’, *Journal of American Medical Association* 311(8), 806–814. 10.1001/jama.2014.732 PMC4770258 24570244

[CIT0010] Pickhardt C, 2012, ‘Adolescence basics’, *Psychology Today*, viewed 16 June 2012, from http://www.psychologytoday.com/basics/adolescence.

[CIT0011] Polit D.F. & Beck C.T, 2008, *Nursing research: Generating and assessing evidence for nursing practice*, 8th edn. Lippincott, Philadelphia, PA.

[CIT0012] Puoane T., Fourie J.M., Shapiro M., Rosling L., Tshaka N.C. & Oelefse A, 2005, ‘“Big is beautiful” – An exploration with urban black community health workers in a South African township’, *South African Journal Clinical Nutrition* 18(1), 6–15. 10.1080/16070658.2005.11734033

[CIT0013] Raj M. & Kumar K.R, 2010, ‘Obesity in children and adolescents’, *Indian Journal of Medical Research* 132, 598–607. 21150012 PMC3028965

[CIT0014] Ramukumba T.S, 2012, ‘A community specific intervention to reduce obesity and related health risks’, D. Tech thesis, Tshwane University of Technology, Pretoria.

[CIT0015] Reddy S.P., James S., Sewpaul R., Koopman F., Funani N.I., Sifunda S. et al., 2010, *Umthente uhlaba usamila – The South African Youth Risk Behaviour Survey 2008*, Medical Research Council, Cape Town.

[CIT0016] Reilly J.J., Methven E., McDowell Z.C., Hacking B., Alexander D., Stewart L. et al., 2003, ‘Health consequences of obesity’, *Archives of Diseases in Children* 88, 748–752. 10.1136/adc.88.9.748 PMC1719633 12937090

[CIT0017] Rossouw H.A., Grant C.C. & Viljoen M, 2012, ‘Overweight and obesity in children and adolescents: The South African problem’, *South Africa Journal of Science* 108(5/6), 1–7. 10.4102/sajs.v108i5/6.907

[CIT0018] Sahoo K., Sahoo B., Choudhury A.K., Sofi N.Y., Kumar R. & Bhadoria A.S, 2015, ‘Childhood obesity: Causes and consequences’, *Family Medicine Primary Care* 4(2), 187–192. 10.4103/2249-4863.154628 PMC4408699 25949965

[CIT0019] Schwarz S.W. & Peterson J, 2010, ‘Adolescent obesity in United States’, *Facts for policymaker*, viewed 06 December 2012, from http://academiccommons.columbia.edu/catalog/ac:135723.

[CIT0020] Silva D.A.S., Pelegrini A., Silva J.M.F. & Petroski A.L, 2011, ‘Epidemiology of abdominal obesity among adolescents from a Brazilian State Capital’, *Journal of Korean Medicine Science* 26(1), 78–84. 10.3346/jkms.2011.26.1.78 PMC3012854 21218034

[CIT0021] Stafford M., Cummins S., Ellaway A., Sacker A., Wiggins R.D. & Macintyre S, 2007, ‘Pathways to obesity: Identifying local, modifiable determinants of physical activity and diet’, *Social Science & Medicine* 65, 1882–1897. 10.1016/j.socscimed.2007.05.042 17640787

[CIT0022] Statistics South Africa, 2011, *Statistics South Africa*, viewed 17 January 2013, from http://www.statssa.gov.za/?page_id=1021&id=city-of-tshwane-municipality.

[CIT0023] Tchernof A. & Després J.P, 2013, ‘Pathophysiology of human visceral obesity: An update’, *Physiological Reviews* 93(1), 359–404. 10.1152/physrev.00033.2011 23303913

[CIT0024] Tzioumis E. & Adair L.S, 2014, ‘Childhood dual burden of under- and overnutrition in low- and middle-income countries: A critical review’, *Food Nutrition Bulletin* 35(2), 230–243. 10.1177/156482651403500210 25076771 PMC4313560

[CIT0025] Van der Wouden J.C., Blankenstein A.H., Huibers M.J.H., Van der Windt D.A.W.M., Stalman W.A.B. & Verhagen A.P, 2007, ‘Survey among 78 studies showed that Lasagna’s law holds in Dutch primary care research’, *Journal of Clinical Epidemiology* 60(2007), 819–824. 10.1016/j.jclinepi.2006.11.010 17606178

[CIT0026] Vanhelst J., Hardy L., Bert D., Duhem S., Coopman S., Libersa C. et al., 2013, ‘Effect of child health status on parents’ allowing children to participate in pediatric research’, *BMC Medical Ethics* 14, 7 10.1186/1472-6939-14-7 23414421 PMC3582492

[CIT0027] Wallace E.V, 2006, ‘The epidemic of obesity in African American communities and the need for a culturally sensitive after school childhood obesity prevention program’, *Californian Journal of Health Promotion* 4(1), 129–133.

[CIT0028] World Health Organization, 2011, *Waist circumference and waist–hip ratio: Report of a WHO expert consultation*, Geneva, 8–11 December 2008, viewed 25 January 2017, from http://www.who.int/about/licensing/copyright_form/en/index.html.

[CIT0029] World Health Organization, 2015, *Maternal, newborn, child and adolescent health*, viewed 15 June 2016, from http://www.who.int/maternal_child_adolescent/topics/adolescence/dev/en/.

[CIT0030] World Health Organization, 2016, *Global strategy on diet, physical activity and health*, viewed 16 June 2016, from http://www.who.int/dietphysicalactivity/childhood_consequences/en/

[CIT0031] Zwiegenthal V., Puoane T., Reynolds L., London L., Coetzee D., Alperstein M. et al., 2009, *Fresh perspectives: Primary health care*, Pearson Education and Prentice Hall: Cape Town.

